# ACL reconstruction postoperative rehabilitation in China: a bibliometrics study and visualization analysis via CiteSpace

**DOI:** 10.3389/fresc.2026.1848419

**Published:** 2026-06-23

**Authors:** Lindan Zhai, Zhiwei Mu, Zhen Wu, Wei Liu

**Affiliations:** Department of Rehabilitation Medicine, GuangZhou Red Cross Hospital of Jinan University, Guangzhou, China

**Keywords:** anterior cruciate ligament, CiteSpace, postoperative rehabilitation, rehabilitation training, rehabilitation treatment

## Abstract

**Objective:**

To analyze the research status, hotspots, and trends in the field of postoperative rehabilitation treatment for anterior cruciate ligament (ACL) reconstruction in China from 2005 to 2025, so as to provide reference for research in this field.

**Methods:**

Taking CNKI and Wanfang databases as data sources, the search terms included “anterior cruciate ligament reconstruction” combined with related terms such as “rehabilitation treatment” and “rehabilitation training”. A total of 1,052 Chinese literatures published from January 2005 to July 2025 were screened. CiteSpace 6.3.R1 software was used for visual analysis of the trend of publication volume, author and institution cooperation networks, and keywords.

**Results:**

The annual number of publications in this field in China showed a fluctuating increasing trend, reaching a peak of 119 in 2024; the density of the author cooperation network was only 0.005, and Ma Yubao was the core author (8 publications); the institution cooperation network was loose, with Sichuan Orthopedic Hospital and other institutions as the main research units; keyword clustering formed 9 core fields including functional training and proprioception, and the research hotspots experienced three stages of evolution: basic rehabilitation (2013–2016), technology integration (2020–2022), and precision rehabilitation (2022–2023).

**Conclusions:**

The research on rehabilitation treatment after ACL reconstruction in China is developing well, but there is insufficient cross-institutional and cross-regional cooperation. In the future, it is necessary to strengthen multi-center collaboration and focus on the research and development of intelligent rehabilitation and precision training technologies.

## 1 Introduction

Anterior Cruciate Ligament (ACL) injury is one of the most common sports injuries of the knee joint. Against the backdrop of China' s nationwide campaign for health and physical activity, the incidence of ACL injury is increasing year by year. As the gold standard for the treatment of complete ACL rupture, ACL reconstruction can restore the anatomical structure of the knee joint, but it may be accompanied by postoperative complications such as knee joint instability, decreased proprioception (1), quadriceps atrophy, changes in lower extremity biomechanics (2, 3), and even long-term secondary injuries to the meniscus and cartilage (4, 5). Therefore, solving postoperative knee joint function problems has become a research hotspot among researchers (6). In addition to clinical rehabilitation contexts, musculoskeletal injury burden and its determinants have also been examined in occupational and applied settings, highlighting the broader importance of injury prevention and rehabilitation strategies (7). Although research in this field has advanced rapidly in recent years, several challenges remain, including the lack of standardized rehabilitation protocols, prominent conflicts between individual heterogeneity and standardized rehabilitation, and the lagging translation of evidence-based research into clinical practice. However, facing a large number of research literatures and rapidly changing research hotspots, how to systematically sort out the research status, grasp the development context, and identify cutting-edge directions in this field has become an important issue to be solved urgently. As an emerging bibliometric analysis method, scientific knowledge mapping can intuitively show the knowledge structure, research hotspots, and development trends of a disciplinary field through visualization technology (8, 9). As a representative tool in this field, CiteSpace software has been widely used in the analysis of research hotspots and prediction of development trends in various medical disciplines. By systematically analyzing the research literatures on postoperative rehabilitation treatment of ACL reconstruction in China in the past 20 years using CiteSpace, it is not only possible to comprehensively understand the research status of this field, but also to provide reference and ideas for clinical practice and future research directions.

## 2 Materials and methods

### 2.1 Data sources

Chinese literatures were searched by title in CNKI and Wanfang databases using the search formula: ‘TI=“Anterior Cruciate Ligament Reconstruction” AND TI=“Rehabilitation Treatment” OR TI=“Rehabilitation Training” OR TI=“Rehabilitation Exercise” OR TI=“Sports Training” OR TI=“Sports Exercise”’. The search language was “Chinese”, and the search time was from January 1, 2005 to July 28, 2025. The literatures were screened to exclude conference papers and duplicate literatures, and a total of 1,052 literatures from CNKI and Wanfang databases were included.

### 2.2 Research methods

Literatures from CNKI and Wanfang databases were exported in “Refworks” format and converted in CiteSpace (6.3.R1) software. The plain-text files were named in the format of “download_xxx”. Finally, the processed information was imported into CiteSpace (6.3.R1) software for visual analysis. Parameter settings: time slicing (2005—2025), 1 year per slice, all “term sources” selected by default, node type (one corresponding type selected each time), K = 8 in g-index, and other parameters set to system defaults.Recent bibliometric studies have similarly applied visualization techniques to map research trends and thematic structures in sports and rehabilitation sciences (10, 11).

### 2.3 Main outcome measures

Each clustering operation generates corresponding Q values and Silhouette values. When the Q value is greater than 0.3, it indicates that the clustering is meaningful; when the Silhouette value is greater than 0.5, it indicates that the clustering has good homogeneity and high reliability (12). Centrality is used to measure the importance of literatures, and nodes with a centrality value exceeding 0.1 are called key nodes (13).

## 3 Results

### 3.1 Temporal distribution of publication volume

**Figure 1:**
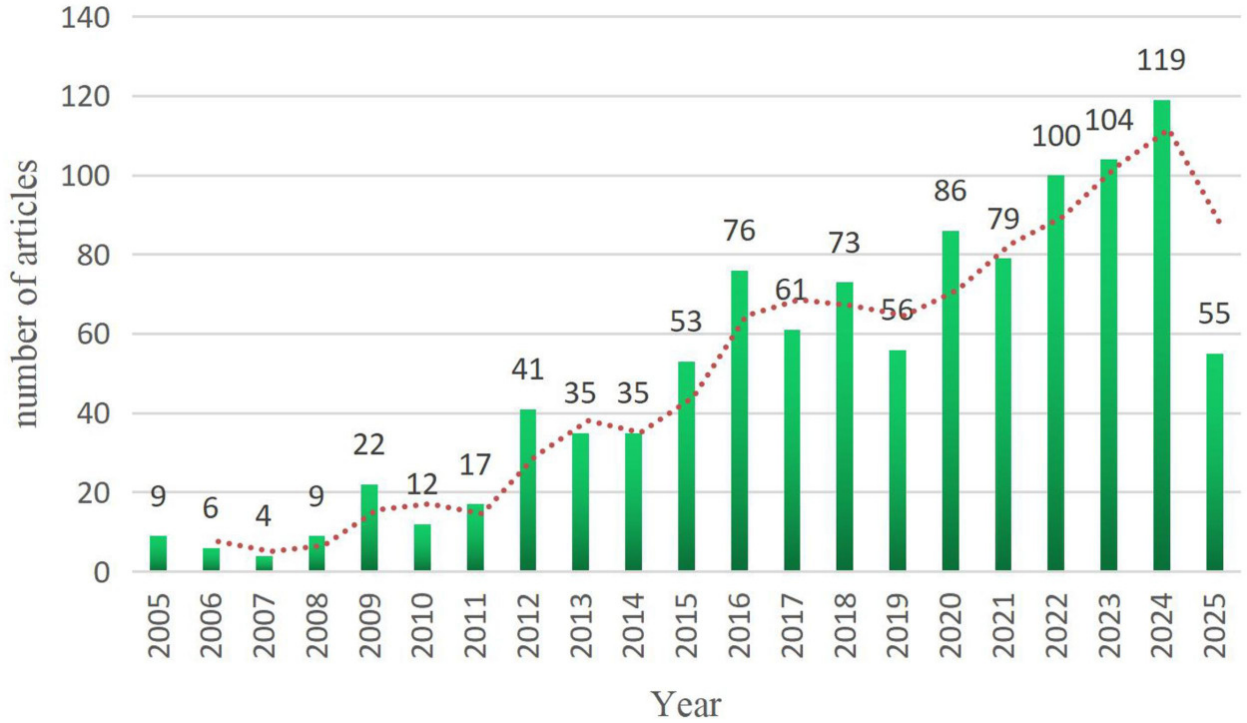
Trend chart of publication volume of postoperative rehabilitation research for anterior cruciate ligament reconstruction in CNKI and Wanfang databases.

A total of 1,052 Chinese literatures were retrieved from CNKI and Wanfang databases, and the annual number of publications showed a fluctuating increasing trend. The maximum annual number of publications was 119 in 2024. The possible reason for the decline in literatures in 2025 is that the full-year literatures were not selected. As shown in Figure 1, the number of research literatures on ACL reconstruction in China has increased steadily in the past 20 years, showing a good development trend.

### 3.2 Analysis of author cooperation network

**Figure 2:**
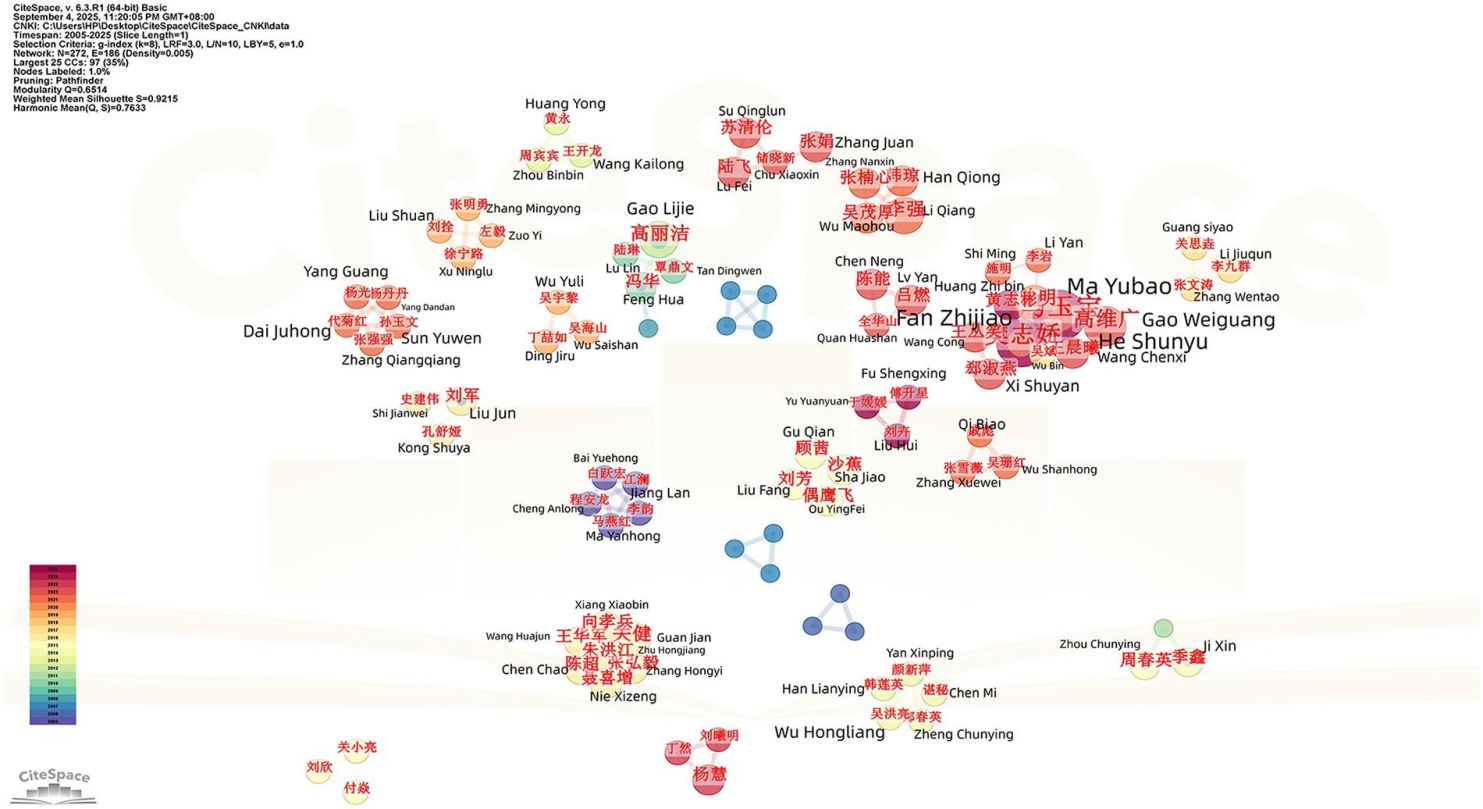
Author cooperation network Map of postoperative rehabilitation research for anterior cruciate ligament reconstruction in CNKI and Wanfang databases.

For the literatures screened from CNKI and Wanfang databases, when “Author Cooperation” was selected as the Node Type and K = 8 in g-index, as shown in Figure 4, there were 272 nodes and 186 connections, with a network density of 0.005. The top 4 authors in terms of publication volume are shown in Table 1. Ma Yubao had the largest number of publications, totaling 8. His most cited literature indicates that neuromuscular training is an important rehabilitation training method after ACL reconstruction, which can improve the neuromuscular control ability of the knee joint, enhance knee joint stability, and promote the recovery of motor ability (Progress in the Application of Neuromuscular Training in Postoperative Rehabilitation of Anterior Cruciate Ligament Reconstruction). As shown in Figure 2, the network density of the author co-occurrence map is relatively low, only 0.005, reflecting that the cooperation between authors is relatively loose and the connection is not close enough.

**Table 1:** Number of publications of the Top 4 authors in postoperative rehabilitation research for anterior cruciate ligament reconstruction in CNKI and Wanfang databases.

Author	Organization	Count
Ma Yubao	Musculoskeletal Rehabilitation Center, Beijing Rehabilitation Hospital Affiliated to Capital Medical University	8
Fan Zhijiao	Rehabilitation Diagnosis and Treatment Center of Beijing Rehabilitation Hospital Affiliated to Capital Medical University	7
He Shunyu	Department of Sports Trauma, Guangzhou Orthopedic Hospital	7
Gao Weiguang	Department of Graduate Work, Shenyang Sport University	5

### 3.3 Analysis of institution cooperation network

**Figure 3:**
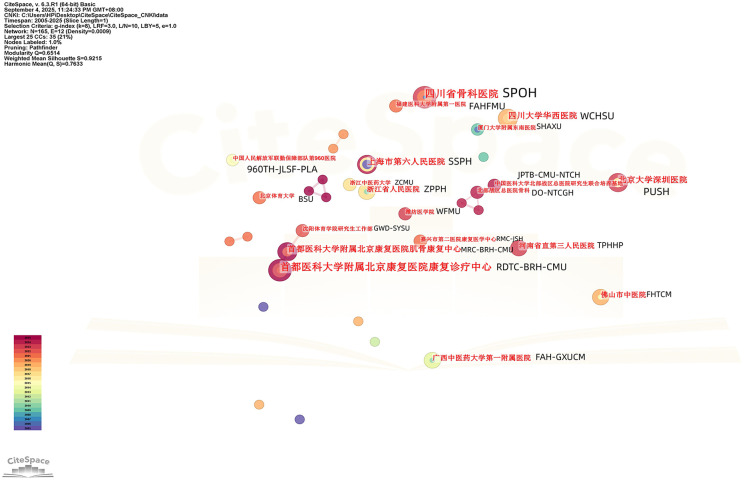
Institution cooperation network Map of postoperative rehabilitation research for anterior cruciate ligament reconstruction in CNKI and Wanfang databases.

For the literatures screened from CNKI and Wanfang databases, when “Institution” was selected as the Node Type and K = 8 in g-index, as shown in Figure 3, there were 165 nodes and 12 connections. Sichuan Orthopedic Hospital and the Treatment Center of Beijing Rehabilitation Hospital Affiliated to Capital Medical University had the largest number of publications, both 6. It can be seen from the figure that the centrality of institutions is generally very low, and institutions in different regions lack cooperation. Table 2 shows the top 4 institutions with the highest number of publications. Due to the extremely small centrality, it can be ignored.

**Table 2:** Institution cooperation network Map of postoperative rehabilitation research for anterior cruciate ligament reconstruction in CNKI and Wanfang databases.

Organization	Count
Sichuan Orthopedic Hospital of Traditional Chinese Medicine	6
Rehabilitation Diagnosis and Treatment Center of Beijing Rehabilitation Hospital Affiliated to Capital Medical University	6
Eastern Theater General Hospital	5
Beijing Rehabilitation Hospital	5

### 3.4 Keyword visualization analysis

#### 3.4.1 Keyword co-occurrence analysis

**Figure 4:**
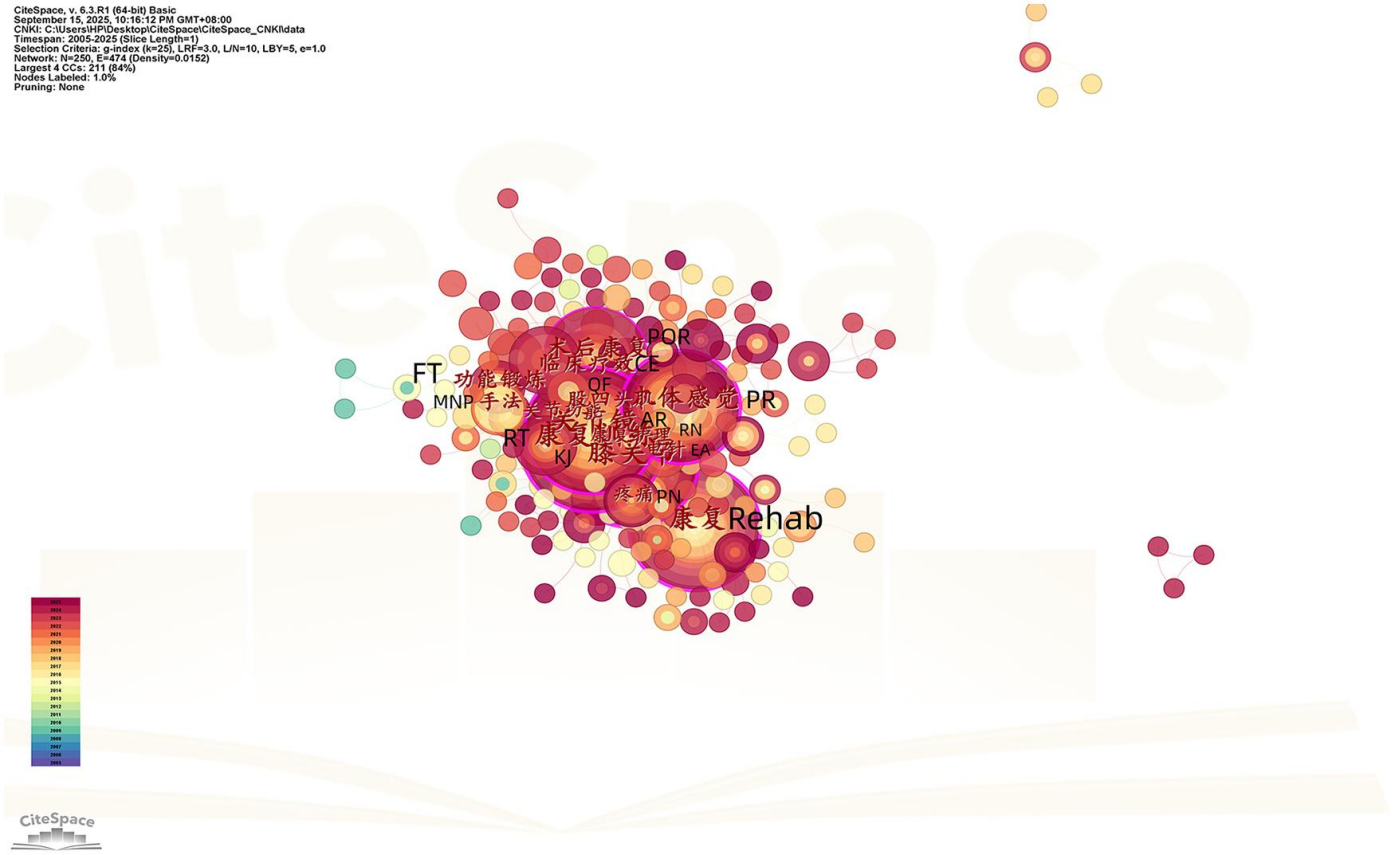
Keyword Co-occurrence Map of postoperative rehabilitation research for anterior cruciate ligament reconstruction in CNKI and Wanfang databases. POR, proprioception; FT, functional training; CE, clinical effects; QF, quadriceps femoris; PR, proprioception; AR, arthroscopes; MNP, manipulation; RT, functional training; RN, rehabilitation nursing; KJ, knee Joint; EA, electroacupuncture; PN, pain rehab:rehabilitation.

The keyword co-occurrence map, as shown in Figure 4, obtained 250 nodes and 474 connections. After excluding subject terms identical to the search formula, the keywords with high co-occurrence frequency are shown in Table 3. By analyzing the co-occurrence frequency and centrality of keywords, it can be seen that the domestic hot keywords mainly include proprioception, early rehabilitation, functional exercise, manipulation, quadriceps, etc.

**Table 3:** Frequency and centrality of postoperative rehabilitation research for anterior cruciate ligament reconstruction in CNKI and Wanfang databases.

Keyword	Frequency	Keyword	Centrality
Rehabilitation training	53	Knee Joint	0.38
Rehabilitation	45	Rehabilitation training	0.32
Arthroscopes	45	Rehabilitation	0.30
Knee Joint	44	Arthroscopes	0.25
Proprioception	33	Proprioception	0.22
Postoperative rehabilitation	27	Postoperative rehabilitation	0.19
Clinical effects	19	Clinical effects	0.10
Early rehabilitation	13	Functional training	0.06
Function of joint	12	Manipulation	0.06
Quadriceps femoris	10	Quadriceps femoris	0.05

#### 3.4.2 Keyword clustering analysis

**Figure 5:**
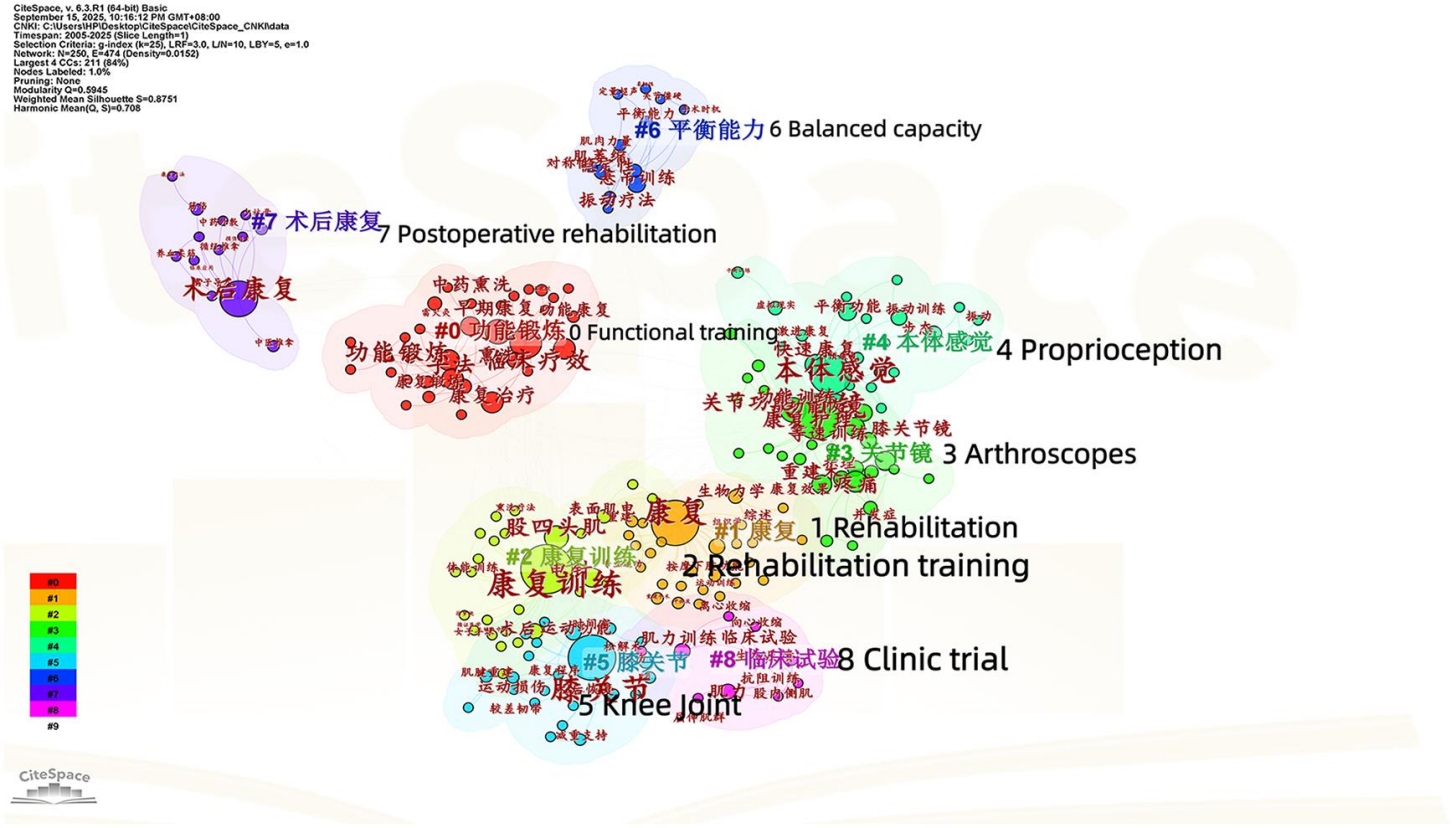
Keyword clustering Map of postoperative rehabilitation research for anterior cruciate ligament reconstruction in CNKI and Wanfang databases.

Keyword clustering groups closely related keywords into one category, so as to have a macro understanding of the current research field. Through LLR clustering analysis of the keywords of the literatures screened from CNKI database, in the obtained clustering map, as shown in Figure 5, the silhouette value S is 0.8751 (> 0.7), indicating good clustering and high homogeneity. The clusters are #0 Functional Training, #1 Rehabilitation, #2 Rehabilitation Training, #3 Arthroscopy, #4 Proprioception, #5 Knee Joint, #6 Balance Ability, #7 Postoperative Rehabilitation, #8 Clinical Trial.Bibliometric evidence also indicates a growing research emphasis on lower extremity injuries and rehabilitation-focused themes in football-related sports, reflecting their clinical and performance relevance (11).

#### 3.4.3 Keyword burst analysis

**Figure 6:**
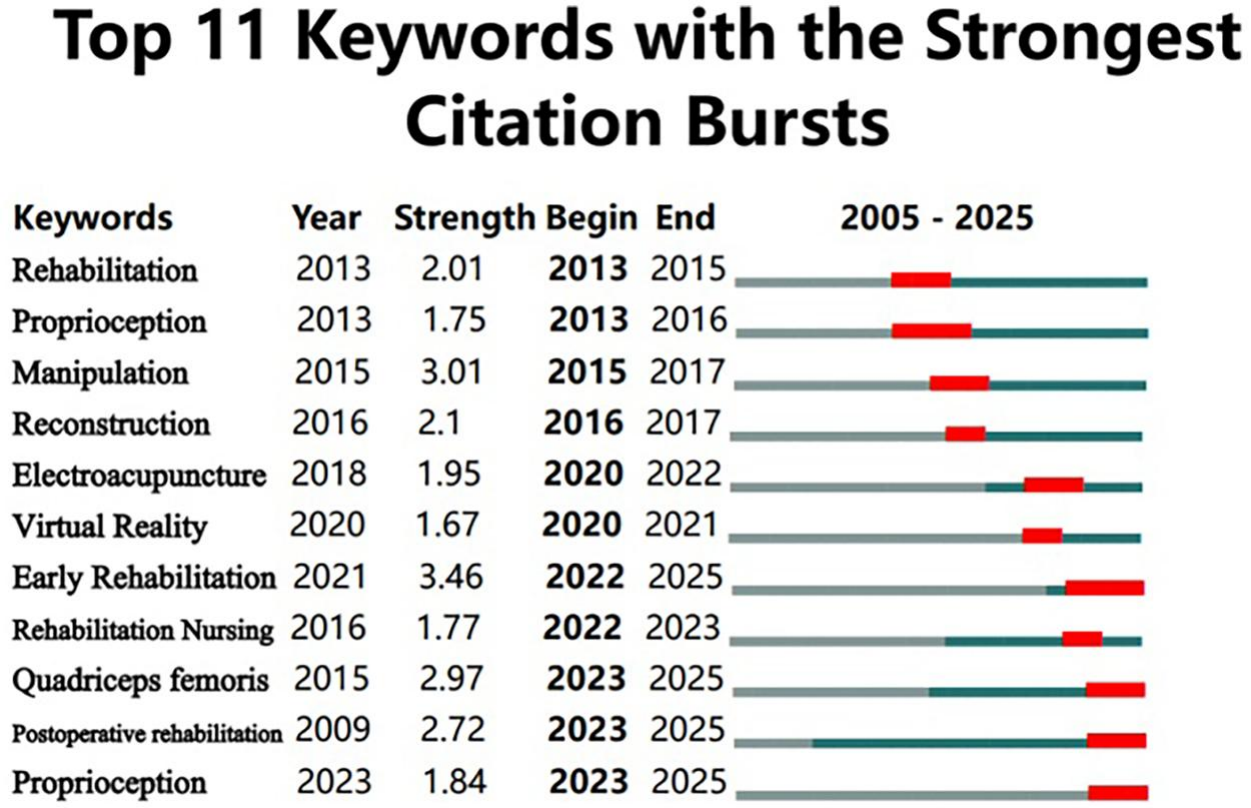
Keyword burst Map of postoperative rehabilitation research for anterior cruciate ligament reconstruction in CNKI and Wanfang databases.

Citation Bursts refer to keywords whose related literatures are cited in large numbers in a short period of time, which can show the research status of this stage and reflect the research hotspots of the next stage. For the literatures in CNKI and Wanfang databases, “Burstness” was selected on the basis of keyword co-occurrence, and CiteSpace V was run to obtain the burst keyword map, as shown in Figure 6. The research on postoperative rehabilitation treatment of ACL reconstruction mainly includes three stages: 2013—2016: rehabilitation, proprioception, manipulation; 2020—2022: electroacupuncture, virtual reality, rehabilitation nursing; 2022—2023: quadriceps, postoperative rehabilitation, balance function.Recent bibliometric mapping studies further confirm the increasing scientific attention toward rehabilitation, performance optimization, and injury prevention in sports sciences (10).

## 4 Discussion

### 4.1 Research status of postoperative rehabilitation training for anterior cruciate ligament reconstruction

From the perspective of annual publication volume, the research on postoperative rehabilitation training after ACL reconstruction has shown an obvious upward trend from 2005 to 2025, which objectively indicates that postoperative rehabilitation training after ACL reconstruction has attracted more and more attention, especially in sports medicine, orthopedics, rehabilitation medicine, etc. Through the analysis of institution and author cooperation, it is found that domestic research institutions have regional limitations, with insufficient cross-regional communication and cooperation, but the publishing institutions are relatively diverse, such as sports universities, research institutes, medical colleges and universities, and their affiliated hospitals. Therefore, in the future, in addition to strengthening cooperation among domestic institutions, domestic research institutions should also enhance contacts with foreign institutions to improve the centrality and visibility of institutions and promote the development of the field of ACL injury research in domestic institutions.

From the perspective of publishing authors, the top 4 authors in China in terms of publication volume are Ma Yubao, He Shunyu, Fan Zhijiao, and Gao Weiguang. Their main research focuses on the effects of different rehabilitation trainings such as blood flow restriction training, whole-body vibration training, virtual reality balance training, and cognitive motor therapy on quadriceps strength, gait analysis, and proprioception in patients after ACL reconstruction (14–22). From the analysis results of the author cooperation network and institution cooperation network, the cooperation between authors is relatively loose, and institutions in different regions lack cooperation. This disperses research resources, making it difficult to form a strong research force and limiting the depth and breadth of research. In the research process, the lack of cooperation may lead to duplicate research and resource waste, and it is impossible to fully integrate the advantages of all parties to jointly solve the problems in rehabilitation treatment.

According to the keyword burst analysis, research on rehabilitation following anterior cruciate ligament (ACL) reconstruction in China has exhibited a distinct phased evolutionary pattern. From 2013 to 2016, the bursting keywords were rehabilitation, proprioception, and manual therapy, which was mainly attributed to inadequate clinical attention to postoperative functional recovery and the widespread adoption of proprioception theory, with rehabilitation interventions dominated by conventional conservative approaches. Between 2020 and 2022, electroacupuncture, virtual reality, and rehabilitation nursing emerged as burst terms, driven by the demands of epidemic prevention and control, policies promoting integrated traditional Chinese and Western medicine, and the concept of enhanced recovery after surgery, thereby shifting the rehabilitation paradigm toward intelligence and perioperative care. Since 2022, quadriceps femoris, balance function, and early rehabilitation have become new research hotspots, signifying a transition from generalized rehabilitation to precise targeted rehabilitation that focuses on quadriceps femoris atrophy, safe early rehabilitation, and high-level motor function recovery, and reflecting the advancement of the discipline toward evidence-based practice, individualization, and high-quality return to sports.

### 4.2 Research hotspots and trends of postoperative rehabilitation training for anterior cruciate ligament reconstruction

Through in-depth analysis of keywords, we found that proprioception, early rehabilitation, functional exercise, etc.are the core hotspots of postoperative rehabilitation treatment for ACL reconstruction in China. Proprioception plays a key role in postoperative rehabilitation after ACL reconstruction. After ACL injury, the proprioception of the knee joint decreases sharply, and proprioception is crucial for the stability and motor control of the knee joint (23). Some scholars have confirmed (24, 25) that balance promotion training has a better effect on the recovery of proprioceptive function compared with conventional rehabilitation training. CUPPONE et al. found that vibration training and hydrotherapy can enhance proprioception through tactile stimulation (26). Wang Jie et al. (27) found that training patients with a crisis simulation stress treadmill training system can significantly improve the proprioception of the affected knee in patients after ACL reconstruction. Some scholars have confirmed (28, 29) that virtual reality balance training is better than traditional balance training in improving knee joint proprioceptive disorders after ACL reconstruction by training patients to perform motor control on an unstable plane. At the same time, in neuromuscular training, different training methods are used to help reshape or enhance the muscle strength around the knee joint, and finally achieve the improvement of proprioceptive neuromuscular control ability (30–32). Early rehabilitation is also a concerned hotspot. Chen Bojian, Liu Jun, etc. showed through animal experiments that early activity has no obvious adverse effect on the tendon-bone healing of the graft, and may even be more beneficial (33).Immobilization after anterior cruciate ligament (ACL) reconstruction results in restricted contraction of the peri-knee muscles and unsatisfactory rehabilitation outcomes. Zhao Weiwei et al. believe that adding proprioceptive training to early BWSTT can accelerate the recovery speed of tendon-bone, increase muscle strength and proprioception, and improve motor function and balance function (34). Early rehabilitation training can reduce pain and prevent joint contracture.Recent studies have also highlighted the growing body of research on recreational and rehabilitation-oriented physical activity for individuals with disabilities, emphasizing the importance of inclusive and adaptive rehabilitation strategies (10).

In summary, certain achievements have been made in the postoperative rehabilitation treatment of ACL reconstruction, but there are still some urgent problems to be solved. First, a unified gold standard for rehabilitation has not yet been established. There is a lack of unified domestic standards for the time, intensity, and method of rehabilitation, which leads to clinical doctors lacking clear guidance when formulating rehabilitation plans. Although the importance of early rehabilitation has been widely recognized, there are still relatively few studies on the effect of early rehabilitation in China. The in-depth research on the specific methods and effects of early rehabilitation is not sufficient. At present, there is a lack of sufficient scientific basis for the optimal combination of various training methods, the timing and intensity of training in early rehabilitation.

### 4.3 Conclusions and prospects

In summary, the number of relevant literatures on postoperative rehabilitation treatment of ACL reconstruction in China has increased year by year in the past 20 years, and proprioception, early rehabilitation, functional exercise, etc., after ACL reconstruction have always been the focus of research. However, there is no unified standard for the time, intensity, and method of rehabilitation. Secondly, the research on the methods and effects of early rehabilitation is not in-depth enough, and there is a lack of scientific basis to optimize early rehabilitation treatment. In addition, the cooperation between authors and institutions is not close enough, and research resources are scattered, making it difficult to form a strong research force and limiting the depth and breadth of research.

In the future, the research on postoperative rehabilitation treatment of ACL reconstruction needs to make continuous efforts in terms of unified standards, multi-disciplinary cooperation, personalized programs, early rehabilitation research, and cooperation mechanisms, so as to improve the level of rehabilitation treatment and improve the quality of life and motor ability of patients.

## Data Availability

The original contributions presented in the study are included in the article/Supplementary Material, further inquiries can be directed to the corresponding author.
